# Urinary infection or radiation cystitis? A prospective evaluation of urinary symptoms in patients submitted to pelvic radiotherapy

**DOI:** 10.6061/clinics/2019/1388

**Published:** 2019-11-19

**Authors:** Vítor Fonseca Xavier, Flávia Carolina Grosso Gabrielli, Karim Yaqub Ibrahim, Mariana Vilela Soares Gomes, Roger Guilherme Rodrigues Guimarães, Edson Abdala, Heloisa de Andrade Carvalho

**Affiliations:** I Departamento de Radiologia e Oncologia, Instituto do Cancer do Estado de Sao Paulo (ICESP), Hospital das Clinicas HCFMUSP, Faculdade de Medicina, Universidade de Sao Paulo, Sao Paulo, SP, BR; II Departamento de Radioterapia, Hospital DF Star, Brasilia, DF, BR

**Keywords:** Neoplasms, Cancer, Radiation Therapy, Pelvic Radiation Therapy, Urinary Tract Infection, Cystitis, Incidence, Cohort Studies, Prospective Study, Controlled Study

## Abstract

**OBJECTIVES:**

The purpose of this study was to evaluate the incidence of urinary tract infection (UTI) in patients with cystitis symptoms who underwent pelvic radiation therapy and identify correlated predictive factors.

**METHODS:**

A prospective cohort study was conducted of patients who met the following: primary pelvic cancer treated with curative intent, older than 18 years old, and good performance status. The exclusion criteria were patients being treated for a UTI, using a urinary catheter, in dialysis or with cystostomy or nephrostomy, and using antibiotics during treatment. Urinalysis and urine culture were collected before the beginning of radiation therapy. Weekly evaluations of urinary symptoms were subsequently performed. In cases of new or worsening symptoms, a questionnaire was applied, and new urine exams were collected. The UTI diagnosis was defined by uroculture as bacterial growth greater than 10^4^ CFU/mL.

**RESULTS:**

From September 2014 to November 2015, 112 patients were sequentially recruited, and 72 (64%) fulfilled the inclusion criteria. During follow-up, 24 (33%) patients had new urinary symptoms or worse preexisting symptoms. A UTI was confirmed in the second urinary culture in only one (1.4%) patient.

**CONCLUSIONS:**

The incidence of UTI was much lower than expected, suggesting that asymptomatic bacteriuria develops symptoms due to radiotherapy. Due to the low rate of UTI, no predictive factor was identified.

## INTRODUCTION

The urinary bladder is frequently exposed, partially or entirely, during radiation therapy for pelvic tumors. Due to the effect of radiation on epithelial cells, acute functional alterations, including increased urinary frequency, dysuria and hematuria, can occur and may mimic the secondary symptoms of urinary tract infection (UTI) (1).

The diagnosis of UTI is typically performed in the general population based on signs and symptoms of cystitis, such as dysuria, polyuria, urinary urgency, suprapubic pain, and leukocyturia or bacteriuria in a biochemical analysis of the urine sample, in the absence of vaginal symptoms in women (2,3). When these findings are observed, empirical antibiotic therapy is frequently initiated, especially in women, while waiting for confirmation by urinary culture (3).

Although this approach has been established for women in the community, it probably cannot be applied in individuals undergoing pelvic radiation therapy. Usually, this population has secondary cystitis symptoms resulting from the cancer or its treatment (4-6). In addition, it has been estimated that alterations observed on urinalysis, such as a high level of leukocytes, a classic predictor of infectious cystitis, may also be present in radiation cystitis (7). Approximately half of patients with localized prostate cancer who underwent radiation therapy with external beam irradiation present urinary symptoms during treatment (5,6,8). However, the estimated incidence of UTI in patients submitted to pelvic radiation therapy varies from 6% to 45% (9-11).

Generally, the assessment of UTI incidence is considered a secondary outcome; hence, no methodology is implemented to exclude biases associated with this diagnosis (12). Thus, there may be a low correlation between cystitis symptoms and the presence of UTI in this population.

Currently, no available evidence is helpful in clinically distinguishing infectious and radiation cystitis. The signs, symptoms and biochemical urine analysis commonly used in the general population to diagnose UTI probably are not applicable in the population submitted to pelvic radiation therapy. This could lead to excessive use of resources, which could generate costs in propaedeutics and compromise patient safety by leading to the excessive use of antibiotics.

The purposes of this study were to evaluate the incidence of UTI among patients with symptoms of cystitis during pelvic radiation therapy and identify possible correlated predictive factors.

## MATERIALS AND METHODS

A prospective cohort study was performed in patients submitted to pelvic radiation therapy. The study was approved by the respective Ethics in Research Committees of the Hospital das Clínicas of the Faculty of Medicine of the University of São Paulo (HC-FMUSP) and the Cancer Institute of the State of São Paulo (ICESP). All patients who agreed to participate in the study signed an informed consent form.

The inclusion criteria were patients with prostate, rectal, anal canal, uterine cervix, or endometrial cancer who underwent pelvic radiation therapy with curative intent and had a performance status less than or equal to 2 (*Eastern Cooperative Oncology Group* - ECOG) (13). Patients using antibiotics or urinary catheters in the 30 days prior to or during radiation therapy and patients on dialysis or with cystostomy or nephrostomy were excluded.

On the day of radiotherapy simulation, urine samples were collected for urinalysis and urine cultures from the eligible patients who agreed to participate in the study. During treatment, urinary symptoms were assessed in weekly routine evaluations with the nursing and medical staff. The specific protocol of the study included an evaluation of the main urinary symptoms related to the treatment, which were graded according to *Radiation Therapy Oncology Group* (RTOG) scores (14). Briefly, urinary symptoms, such as urinary frequency, nocturia, dysuria, and urgency, were graded from 0 (zero) or none to 4 (worse). The patients were followed up during treatment. In the event of the appearance of urinary symptoms or worsening of preexisting symptoms, a questionnaire (Appendix) was applied to analyze possible risk factors, and a second urinalysis and culture were performed. The diagnosis of UTI was established when a urinary culture showed bacterial growth greater than 10^4^ CFU/mL (*Gelose chromID^®^*).

### Statistical Analysis

Data in the literature show that the incidence of dysuria is approximately 50% among patients submitted to pelvic irradiation and that the rate of UTI varies from 6% to 45% in this population (7-10). The sample size of this study was calculated based on a 50% incidence of dysuria and a 10% incidence of UTI. Considering that approximately 10 cases per variable were needed for the multiple regression analysis, five variables were pragmatically chosen for the analysis. Thus, 50 events of UTI would be necessary so that an initial sample of 1,000 patients, which was calculated to contain at least 500 symptomatic patients was required.

Descriptive and frequency analyses were used to describe and systematize all the data obtained during this study. A multivariate regression analysis was planned to identify factors that could predict the risk of infection and provide early guidelines for clinical outcomes.

## RESULTS

From September 2014 to November 2015, 112 patients were recruited, and an interim analysis was then conducted. Among the recruited patients, 29 were excluded because they did not perform the first requested urinalysis type I exam and uroculture, as were 11 patients who had a first positive urinary culture. Thus, a total of 72 patients were included and evaluated. Of these, 24 (33%) presented urinary symptoms during irradiation, only one (4%) of whom had a positive urinary culture.

Due to the lower than expected incidence of symptomatic patients and especially the low incidence of UTI found in the study, which was much lower than that used for the sample calculation, patient recruitment was interrupted, and the initially proposed regression analysis of UTI-predictive factors was aborted.

The patients included in the study were mostly male (60%) and older than 40 years old (98%). Prostate and rectal cancers were more prevalent, at 36% and 31%, respectively. The proportions of asymptomatic (51%) and symptomatic (49%) patients were similar before treatment. Urinary urgency was the most reported symptom (63%). Table 1 shows the patients’ characteristics.

During radiation therapy, only 24 (33%) patients presented symptoms. The 48 patients who remained asymptomatic were not submitted to any further urine exams. Patients with primary anal canal cancer presented a higher rate of symptoms (67%; 2/3 patients). Approximately one-third of the patients with primary uterine cervix cancer, prostate cancer, and rectal cancer also presented symptoms, while the four patients with endometrial tumors did not present any symptoms (Table 2).

Regarding the treatment technique, all patients received external beam irradiation in the pelvis, and only 13 (18%) patients with gynecological tumors underwent intracavitary brachytherapy. The doses of external beam irradiation ranged from 45 Gy to 78 Gy in fractions of 1.8 to 2 Gy/day. Brachytherapy doses were 20 Gy across 4 fractions of 5 Gy each on the vaginal vault surface (postoperative treatment) or 30 Gy across 4 fractions of 7.50 Gy each at point A for definitive treatment.

Among the patients who presented symptoms, 29% were treated with tridimensional conformal radiotherapy (3D-CRT), 50% with intensity-modulated radiotherapy (IMRT), and only three patients were submitted to brachytherapy (Table 2). In 75% of the patients, the beginning or worsening of the preexisting symptoms started after a median dose of 32.4 Gy (range, 15 to 45 Gy) of external beam radiotherapy. The great majority of the patients (83%) presented dysuria as the symptom that required the second urinary culture. No hematuria or urinary urgency was reported (Table 2).

## DISCUSSION

This study was proposed and designed to provide the maximum information regarding urinary symptoms and infection in the population submitted to pelvic radiation therapy. The study design was established to control possible biases related to the definite diagnosis of UTI through positive urinary culture. The aim of this study was to provide guidance for propaedeutics and therapeutics of these patients.

The estimated incidence of acute urinary symptoms in the population submitted to pelvic radiation therapy is widely variable. Values between 25% and 83% are usually described (5,6,10,12,16). A 50% incidence was initially considered for the sample calculation. In our study, 33% (24/72) of the patients presented symptoms, a value that was lower than expected. We attribute this relatively low incidence to the fact that patients were treated with 3D-CRT or IMRT, which are better than conventional bidimensional treatments at normal tissue-sparing (6).

At the 14^th^ month of recruitment, a low number (24) of symptomatic patients was observed, and after an interim analysis, enrollment was interrupted. Thus, it was not possible to perform the evaluation of possible predictive factors for UTI that was originally planned. Therefore, we evaluated the data in a descriptive way.

The higher incidences of prostate and rectal cancers among males and uterine cervix cancer among females in our country (2) reflect the prevalence of these tumors in the study population.

Paradoxically, patients submitted to IMRT, a technique that allows better normal tissue sparing with less toxicity than is caused by 3D-CRT (1,16), presented a higher incidence of symptoms. However, in our daily routine, this technique is reserved for cases of anal canal cancers, patients who need higher doses for tumor control (such as those with prostate cancer), and those with unfavorable anatomy for the sparing of surrounding organs at risk (e.g., bladder cancer) with 3D-CRT.

In our study, only patients with gynecological tumors underwent intracavitary brachytherapy. It seems intuitive that these patients would be more prone than other to develop urinary symptoms due to the high dose of irradiation applied to the region near the bladder as well as the need for a bladder catheter during each procedure (7). However, compared to other techniques, these patients had a lower incidence of symptoms, at only 3/13 (23%). The other 10 patients underwent treatment without urinary complaints.

In accordance with the average lifetime of mature cells of the bladder and urethral epithelium (9), new symptoms or the worsening of preexisting ones were observed in patients who received a median dose of 32.4 Gy after approximately 2 to 3 weeks of radiation therapy.

Urinary symptoms resulting from radiation therapy are usually reported on a generic genitourinary toxicity scale, such as an RTOG score (14). Dysuria was the most reported symptom and triggered a request for the second examination in 83% of affected cases, resulting in an incidence of 28% among treated patients (20/72).

At least two recent studies have prospectively assessed the incidence of UTI among the population undergoing pelvic radiation therapy. Both aimed to evaluate the effects of cranberry ingestion in controlling cystitis symptoms and the incidence of UTI. Bonneta et al. (10) evaluated 370 patients who underwent pelvic radiation therapy and found a UTI incidence of 24.2%. Cowan et al. (15) performed a randomized trial with 128 patients who took a placebo or cranberry. The incidence of UTI among this population was 44.1%. Although the incidence of UTI was high in these studies, no urinary evaluation was performed before treatment. The absence of a urinalysis control before the beginning of treatment may have contributed to the high incidence of UTI among the study population as several of these patients could have been carriers of asymptomatic bacteriuria.

Only two other studies have performed a prospective evaluation of the population involving type I urinalysis and urine culture in all patients prior to treatment. Bialas et al. (9) evaluated 172 patients in 1989 and prospectively observed that the prevalence of positive uroculture was 17% prior to radiotherapy and 17% during treatment. In a 1994 study, Prasad et al. (11) prospectively evaluated patients with primary gynecological tumors. They found that the risk of UTI was higher among those submitted to cystoscopy prior to radiation therapy and bladder catheterization in intracavitary brachytherapy. Weekly urinary cultures were performed without symptom assessment, and 10% of the patients presented with a positive urinary culture before the beginning of treatment, while 23% presented with positive results during radiation therapy.

In their study, Shuford et al. (7) evaluated the urine exams of 134 women with primary pelvic tumors treated with pelvic radiation therapy. The purpose was to identify urine analysis parameters that could predict UTI with urinary culture used as the gold standard. However, theirs was a retrospective study without an assessment of urinary status prior to the beginning of treatment. Therefore, their results may contain the same biases found in previous studies regarding patients with asymptomatic bacteriuria.

In our study, all 112 of the evaluated patients underwent urinary culture prior to the beginning treatment, and 10% were identified as already having a positive urinary culture, a value close to those of the previous 2 studies that performed this evaluation prior to treatment. We decided to exclude these patients in advance because we sought to evaluate the actual incidence of UTI during treatment.

When we compared the incidence of UTI during treatment obtained in this study to those obtained in previous studies that included a control urinary culture performed prior to the beginning of radiation therapy, we found that ours was much lower. While Bialas et al. and Prassad et al. reported incidences of 17% and 23%, respectively, ours was only 4% (one patient). This difference may be because the two previous studies performed weekly urinary culture exams in all patients regardless of the presence or absence of symptoms.

We consider, therefore, that the 4% incidence of UTI detected in our study is well below that found in the previous literature mainly due to two factors. The first factor is that we performed urinary culture exams before radiation therapy and excluded patients with positive results. The second is that we investigated only patients with urinary symptoms. Thus, we excluded patients with asymptomatic bacteriuria and investigated only those who became suspicious for UTI due to the presence of symptoms.

Based on these results, we conclude that the frequency of UTI reported to date actually reflects the incidence of patients with asymptomatic bacteriuria who developed secondary symptoms due to radiation therapy and were erroneously classified as having a UTI based on the classic criteria used for patients in the community.

Our results are extremely important for clinical practice. The low incidence of UTI found in our study should prompt a discussion about the application of the clinical diagnostic criteria for UTIs used in patients undergoing pelvic radiation therapy. The frequency of false diagnoses appears to generate excessive empirical treatments in this population.

To the best of our knowledge, this is the first prospectively controlled study to analyze the real incidence of UTI among the population treated with pelvic radiation therapy. The very low incidence found in this study highlights the importance of using the correct approach in these patients to avoid excessive diagnoses, expenses related to propaedeutics and the application of unnecessary empirical treatments in this population, which can even lead to antimicrobial resistance. Our results should not only stimulate efforts aimed at avoiding empirical treatments, such as antibiotics, in this population but also demonstrate that it is safe to wait for the results of uroculture analysis before selecting the appropriate antibiotic therapy in patients without neutropenia and fever.

## AUTHOR CONTRIBUTIONS

Xavier VF was responsible for the study conceptualization, data curation, formal analysis, investigation, methodology and project administration. Gabrielli FCG was responsible for for the study conceptualization, methodology and formal analysis. Ibrahim KY was responsible for the study conceptualization, methodology and forma analysis. Gomes MVS was responsible for the study conceptualization, data curation and investigation. Guimarães RGR was responsible for the study conceptualization, data curation and investigation. Abdala E was responsible for the study conceptualization and methodology. Carvalho HA was responsible for the study conceptualization, data curation, formal analysis, investigation, methodology and project administration.

## APPENDIX

**Table:** Clinical questionnaire

PATIENT IDENTIFICATION
NAME: AGE:
DATE:
RGHC: PACS:
**SIMULATION TOMOGRAPHY**
URINARY SYMPTOMS: ____Dysuria ____Urinary urgency ___Nocturia (____number) ___Hematuria **EXCLUSION CRITERIA?** ____YES ___NO (if YES, specify) ____ USE OF ANTIBIOTICS IN THE PAST 30 DAYS ____ECOG ≤ 2 ___PALLIATIVE ____ DIALYSIS ____CYTOSTOMY/NEFROSTOMY____URINARY CATHETERS ____ NOT JOIN ____ OTHERS: ______________________________
**NURSE**
**SYMPTOM IDENTIFICATION (DATE)**______/______/______
**ANTIBIOTIC DURING TREATMENT?** ___YES ___NO (if YES, exclude)
**RISK FACTORS**
FECAL INCONTINENCE? ____YES ___NO
DIAPER USE LAST WEEK ___YES ___ NO
UTI NUMBER IN THE LAST 12 MONTHS: __________
LAST UTI TREATED (HOW MANY MONTHS AGO)? _____
CHANGE IN URINE ASPECT IN THE LAST WEEK? ____YES ___NO
DO YOU THINK YOU HAVE A URINARY TRACT INFECTION? ____YES ____NO
URINARY TRACT SYMPTOMS (*Acute Radiation Morbidity Scoring Criteria,* RTOG)
______**GRADE 1** (Does not require medication) ____Dysuria ____Urinary urgency ____Nocturia (require medication)
______**GRADE 2** (requires medication) ____Dysuria ____Urinary urgency ____ Nocturia (2X greater than pretreatment)
______**GRADE 3** (Opioid use for urinary symptoms, macroscopic hematuria, severe nocturia) ____Dysuria ____Urinary urgency ____ Nocturia (1/1 h) ___Hematuria
______**GRADE 4** (Blood transfusion secondary to hematuria, urethral obstruction, necrosis/ulceration)
**PHYSICIAN**
**PRIMARY TUMOR SITE:** ___ PROSTATE___ CERVIX ___ ENDOMETRIUM ___ RECTAL ___ANAL ___Other: Which:______________________
**CONCOMITANT CHEMOTHERAPY?** ___YES ___NO
**RT DOSE ON DATE OF SYMPTOMS:** ________cGy ____Fx
**PRESCRIBED DOSE:** _________cGy ____Fx
**BRACHYTHERAPY BEFORE SYMPTOMS?** ___YES ___NO. IF YES, HOW MANY?______
**LEUKOCYTURIA:** _______________ **HEMATURIA:**____________**NITRITE:**_____________

## Figures and Tables

**Table 1:** Characteristics of the 72 included patients.

Characteristics		Number of patients (%)
Sex	Male	43 (60%)
	Female	29 (40%)
Age	<40 years old	2 (2%)
	40-70 years old	41 (57%)
	>70 years old	29 (42%)
Primary tumor site	Anal canal	3 (4%)
	Cervix	16 (23%)
	Endometrium	4 (6%)
	Prostate	27 (36%)
	Rectum	22 (31%)
Urinary symptoms before radiotherapy	Asymptomatic	37 (51%)
	Symptomatic	35 (49%)
	Dysuria	5 (14%)
	Urgency	22 (63%)
	Nocturia (>2x)	13 (37%)
	Hematuria	0 (0%)

**Table 2:** Characteristics of the 24 (33%) symptomatic patients and their respective symptom incidences during radiation therapy.

Characteristics		Symptom Incidence (%)
Primary tumor site	Anal canal	2/3 (67%)
	Cervix	5/16 (31%)
	Endometrium	0/4 (0%)
	Prostate	10/27 (37%)
	Rectum	7/22 (32%)
Treatment technique	3D-CRT	16/56 (29%)
	IMRT	8/16 (50%)
	Brachytherapy	3/13 (23%)
Pelvic Radiation Dose at symptom appearance/worsening	<5 Gy	0/24 (0%)
	6-15 Gy	2/24 (8%)
	16-25 Gy	6/24 (25%)
	26-35 Gy	6/24 (25%)
	36-45 Gy	6/24 (25%)
	46-55	1/24 (4%)
	56-65 Gy	2/24 (8%)
	>65 Gy	1/24 (4%)
Urinary symptoms	Dysuria	20/24 (83%)
	Urinary urgency	0/24 (0%)
	Nocturia	4/24 (16%)
	Hematuria	0/24 (0%)
